# Greenselect Phytosome for Borderline Metabolic Syndrome

**DOI:** 10.1155/2013/869061

**Published:** 2013-11-14

**Authors:** Gianni Belcaro, Andrea Ledda, Shu Hu, Maria Rosa Cesarone, Beatrice Feragalli, Mark Dugall

**Affiliations:** Department of Biomedical Sciences, Irvine3 Circulation/Vascular Labs & San Valentino Vascular Screening Project, Gabriele D'Annunzio University, Chieti, Italy

## Abstract

The beneficial effects of Greenselect Phytosome, a proprietary lecithin formulation of a caffeine-free green tea catechin extract, were evaluated in a controlled registry study on 50 asymptomatic subjects borderline for metabolic syndrome factors and with increased plasma oxidative stress. After 24 weeks of intervention, improvement in weight, blood lipid profile, and blood pressure positioned 68% of subjects in the treatment arm out of the metabolic syndrome profile, while 80% of the subjects in the control group still remained in their initial borderline disease signature. Compared to the control (lifestyle and dietary changes alone), Greenselect Phytosome was especially effective for weight/waist changes. These results highlight the relevance of addressing multiple factors involved in the development of metabolic syndrome with a pleiotropic agent capable of improving the beneficial effects of lifestyle and dietary changes and foster the attainment of a globally improved health profile.

## 1. Introduction

Tea is probably the most consumed beverage in the world after water, and its pharmacological potential has been extensively investigated [1]. The phytochemical profile of tea depends on the extent of oxidation (*fermentation* in the tea lingo) of its catechin constituents, and most studies have focused on green tea that maintains the original polyphenolic profile of the leaves and is the source of sinecatechins, a registered drug for the topical management of genital warts [1]. Cancer prevention [2, 3], weight loss [4, 5], and diabetes [6–9] are some of the other hot areas of clinical investigation where green tea catechins have proved potentially useful both as a stand-alone agent and in combination with lifestyle and pharmacological intervention strategies. 

According to an estimation from 2010 [10], more than 35% of adults and 17% of children and adolescents are medically obese (BMI > 30) in North America, and obesity rates are rapidly increasing not only in affluent Western countries but also in poorer nations, presumably because of the growing popularity of the obesogenic Western lifestyle [11, 12]. Green tea extracts enriched in catechins have shown promising results in reducing body mass index in over-weight subjects, especially when associated with lifestyle changes and diet [13–18]. A synergistic effect of catechins and caffeine on reducing obesity has been observed, presumably due to a complementary increase in adrenergic tone and its associated signal translation in adipose tissue [19], but the detrimental effects of caffeine on glucose control and blood pressure make this association questionable in the context of overall prevention of the metabolic syndrome (MetS) [20]. Thus, obesity is often associated, especially in adults, with detrimental changes in glucose control and variously related cardiovascular risk factors (dyslipidemia, hypertension). Epigallocatechin gallate (EGCG), the major catechin of green tea, is a well-known thermogenic agent [21] that, unlike caffeine, also shows beneficial effects toward several markers of the metabolic syndrome, especially glucose control and dyslipidemia [22].

Greenselect Phytosome (GSP) is a green tea extract devoid of caffeine and formulated in lecithin to improve the absorption of catechins [23]. Because of its domesticated profile compared to other green tea extracts in terms of phytochemical profile and bioavailability, it was selected, in the form of coated tablets (Monoselect Camellia, MonCam), for a single blind, controlled study on 50 asymptomatic subjects borderline for all five MetS factors, who were also showing an increased plasma oxidative stress. According to the new International Diabetes Federation (IDF) definition [24], metabolic syndrome patients are featuring central obesity as well as two (or more) of the following four factors:

- raised triglycerides level: ≥150 mg/dL (1.7 mmol/L) or need for treatment for this lipid abnormality;
- reduced HDL cholesterol: <40 mg/dL (1.03 mmol/L) in males and <50 mg/dL (1.29 mmol/L) in females or need for treatment for this lipid abnormality;
- raised blood pressure: systolic BP ≥ 130 or diastolic BP ≥ 85 mm Hg or treatment of previously diagnosed hypertension;
- raised fasting plasma glucose: FPG ≥ 100 mg/dL (5.6 mmol/L) or previously diagnosed Type II diabetes.

MetS is associated with an increased risk of cardiovascular events (i.e. coronary disease, myocardial infarction, and stroke) and to the development of type-2 diabetes [25–28]; its management should be aggressive, constant, and effective, involving primary intervention (lifestyle modification with calorie restriction, increase of physical activity, and change in dietary composition) to achieve a 10% loss of body weight in the first year. Even a modest weight loss is associated with a significant risk reduction for Type II diabetes and cardiovascular disease [29–31]. When lifestyle change is not possible or not adequate and in subjects considered at higher risk for cardiovascular disease, a secondary intervention [32–34] resorting to drug therapy may be required to treat MetS, and there is a dire need of agents to control its risk factors. However, since the actual molecular mechanism(s) underlying the development of MetS are still largely elusive, no specific pharmacological agents are currently available to globally control the cardiovascular and diabetic risk associated to MetS [35–37]. The relevance of each single factor in the development of metabolic syndrome is indeed difficult to evaluate and might even be different in distinct populations [32, 38]. In the present study, we wanted to evaluate whether GSP is able to support the beneficial effects of lifestyle and dietary changes and foster the attainment of a globally improved health profile, thanks to its pleiotropic effect able to address multiple factors involved in the development of metabolic syndrome.

## 2. Materials and Methods

### 2.1. Subjects

Inclusion criteria were an asymptomatic status, a borderline profile for all the 5 MetS factors, and an increased oxidative stress. Exclusion criteria for subjects were fasting blood glucose values exceeding 162 mg/dL and any other metabolic or clinical conditions requiring medical treatment. Pregnant or breastfeeding females, individuals with any clinical condition, psychiatric disorders including depression, and subjects who participated in another study less than 30 days before the start of this study were excluded as well.

### 2.2. Study Design

The present study was randomized, single-blinded, parallel design comparing GSP tablets versus a corresponding blank formulation, in association with lifestyle and dietary interventions. 

### 2.3. Study Protocol

At inclusion, subjects were unaware of the presence of MetS and of the value of the MetS factors. The study was performed according to the Declaration of Helsinki, and all patients gave an informed consent prior to their participation to the study. After briefing and evaluation of their interest to participate in the study, the volunteers were divided into two groups using the same management plans.

- Group 1: management plan + MonCam (1 tablet at 10 AM and one at 6 PM) equivalent to overall 300 mg/day of GSP.
- Group 2: management plan + corresponding blank formulation.

Volunteers had a defined management plan: changes in diet, thus avoiding junk food and limiting high calorie elements, and a precise individual exercise plan. Diet produced a caloric deficit (750 kcal/day in females and 1000 kcal/day in males) compared to the individual estimated energy requirements and contained 17–22% proteins, 23–25% fats, and 55–58% carbohydrates. Individual exercise plan foresaw 210 minutes/week of physical activity (70% moderate aerobic, 30% muscular strengthening). None of the included subjects had important (at ultrasound scans) atherosclerotic lesions (plaques or intima-media thickening) in the studied arteries (carotids, femoral, aorta). No other nutritional elements, vitamins, or drugs were used in the observation period. No other clinical condition was present.

Based upon published data about the efficacy of lifestyle changes on MetS [39, 40], an estimate of not less than 25 subjects completing the study in each group was considered necessary to obtain clinically meaningful data on the treatment outcome. The safety population, as planned in the protocol, was effectively composed of all the subjects recruited into the study, who received at least one dose (product or blank formulation) and having at least one post baseline safety evaluation (one week). The intention-to-treat (ITT) efficacy analysis included all the subjects who had received at least one dose of medication and had had at least one post baseline measurement of the primary efficacy items. Subjects having treatment compliance <70% were excluded. The ITT analysis was performed comparing “all treated subjects” versus control subjects. The per-protocol (PP) analysis had to include all subjects available for the ITT analysis, who completed the planned duration of treatment, were complying with the regimen, had a valid efficacy evaluation, and did not violate the protocol in any way liable to influence the efficacy outcome. A range of compliance of at least 85% was adopted as a criterion to select subjects for the PP analysis. Therefore, subjects with compliance <85% were excluded, as well as all the subjects who did not meet the criteria for the “Population for efficacy analysis.” Additional (“post-hoc”) analysis was made to assess the minimum effective time “dose” to reach significant results. These tests were performed using closed testing procedures. The fixed-sequence Dunnett test was applied to determine the time needed to have results on each MetS item. All hypothesis tests were two sided. All statistical tests (nonparametric) were performed at a 5% significance level (*P* < 0.05).

### 2.4. Products

Both the ingredient Greenselect Phytosome (GSP) and its oral formulation in tablets Monoselect Camellia (MonCam) are commercially available products from Indena (Milan, Italy) and from PharmExtracta (Pontenure, Italy), respectively. GSP is a decaffeinated, standardized extract from green tea having the following specifications:

- epigallocatechin-3-O-gallate (EGCG) ≥ 13%;
- polyphenols 19%–25%;
- caffeine ≤ 0.1%.

Each MonCam tablet has been manufactured to contain 150 mg of GSP. No side effects were reported during the execution of the study, formulation tolerability (both for GSP and the corresponding blank formulation) being excellent, with overall more than 93% of GSP tablets and more than 91% of blank formulation having been correctly used.

### 2.5. Blood Tests

At inclusion and at 24 weeks (end of observation), a panel of additional blood tests was carried out (ALT, AST, gamma-GT, Alkaline Phosphatase, CRP, Serum creatinine, Leptin as well as blood cell count and fibrinogen, aPTT, and PT-Quick's value and hematocrit).

### 2.6. Anthropometric Measures

At inclusion and at 24 weeks (end of observation) the following anthropometric parameters were taken in all subjects: body weight, body mass index (BMI), and waist circumference.

### 2.7. Data Analysis

The clinical efficacy assessed by weight and blood test score was examined using a B-W-Subject Anova model, a mixed analysis of variance design for differences mean, which is the only model that can be correctly applied to the experimental data [41]. Analysis of difference between active and blank formulation groups was performed by Tukey-Kramer test for pairwise comparisons between means.

## 3. Results and Discussion

We have been interested in the possibility to improve the outcome of MetS primary prevention by complementing lifestyle changes with dietary ingredients capable to address the various dysfunctions in which the syndrome is declined, and, as discussed above, green tea catechins show indeed the potential to do so. To explore this enticing possibility, we hence carried out a single blind controlled study on 50 asymptomatic subjects borderline for all the five mentioned MetS factors and also showing increased plasma oxidative stress. The study lasted 24 weeks, and 48 (25 men and 23 women) out of 50 subjects in the treatment group completed the study, with no dropouts being due to medical reasons; 50 comparable control volunteers were also followed for all the duration of the study, with no dropouts. The groups characteristics are summarized in Table 1.

The results, which are detailed in Table 2, confirmed the efficacy of the management plan to achieve an overall amelioration, both at the anthropometric and blood parameters level. Compared to the control group, though, who took the blank formulation, the group who took MonCam performed remarkably better, achieving for all the parameters observed a better outcome, statistically significant both versus the baseline and versus the control group, even for items where the control group did not obtain a significant result (waist circumference in women, HDL cholesterol, blood pressure, and fasting glucose).

The panel of additional blood tests did not show any significant variations (within the normal range at inclusion and after the evaluation period, as may be expected in borderline volunteers). 

GSP clearly promoted weight loss and reduced waist circumference (see Table 2) when compared to management plan alone. The improvement in GSP subjects in borderline MetS was also associated with lipids and blood pressure beneficial variations. Finally, the value of plasma free radicals (PFR) decreased at 24 weeks in the GSP group (−33.04%; *P* < 0.05 versus inclusion) in a much more marked way compared to the control (decreasing only −4.14%; n.s. versus inclusion, *P* < 0.022 between groups).

Overall, GSP appears to have a moderate, yet significant, action on each single parameter of MetS, with particular emphasis on weight/waist changes. It was also interesting to evaluate the global impact of the treatment protocol (lifestyle + GSP versus lifestyle + blank formulation) on the various parameters defining metabolic syndrome (Table 3). In accordance with the inclusion criteria, 100% of subjects had borderline values for all the five parameters of MetS. At the end of the study, 68.75% of GSP subjects (33 out of 48) were outside the metabolic syndrome profile, 7 had reached a limit profile for MetS, while only 8 were still lingering in MetS area. Conversely, in the control group, 80% of subjects were still in MetS area at 24 weeks and 10 out of 50 (20%) had still a borderline profile for MetS. Therefore, a significant improvement in all risk conditions and a more significant progressive exit from the MetS area were observed in the GSP branch. In the ITT analysis, 40 out of a total 48 GSP subjects (83.33%) improved in comparison with 20% of controls (10/50), a statistically significant (*P* < 0.02) difference.

## 4. Discussion

This study confirms GSP's efficacy in weight loss when associated with proper lifestyle modifications and a low-calorie diet [18]. This result was achieved despite the absence of caffeine in the product, thus going beyond results found with caffeine-containing green tea extracts [19, 22] and confirming that catechins alone can have an important role in controlling weight, most likely through the increase of fat oxidation [21]. Spurred by epidemiological data, some investigations have been undertaken to evaluate the usefulness of green tea and green tea extracts in MetS [42, 43]; for the time being, the heterogeneity of the studies available in literatures and their still limited numbers makes it difficult to compare our results with those obtained in other studies, although featuring coherent outcomes.

In terms of body weight specifically, it is worth mentioning that the results described above were obtained treating volunteers with a dose of GSP corresponding to 40 mg/day catechins, while other studies on green tea and green tea extracts in weight loss area (although in a different type of population) were done treating volunteers with much higher doses of green tea catechins (375–1200 mg/day) typically associated with caffeine (150–600 mg/day) [15, 44–48]. While the exact composition of green tea infusions and green tea extracts used in the studies available from the literature cannot always be clearly identified, we assume that the achievement of a significant weight loss resorting to a catechins intake which is significantly lower than in other studies can be explained by an improved bioavailability provided by the lecithin component of GSP [49]. Also, the use of a standardized, reproducible extract, as in the case of GSP, allows making future comparisons in case of additional clinical experiences in weight loss and MetS. 

Another important aspect to be evaluated when comparing studies obtained with different green tea derivatives is the presence of caffeine, in addition to the content, composition, and bioavailability of catechins. The role of caffeine is, in fact, double-edged: if, from one side, it can increase the metabolic rate and calorie expenditure [50], on the other side, it is able to disrupt insulin sensitivity at low doses even in healthy volunteers [51], possibly leading to an undesirable weight loss outcome as a result of these two opposite effects. Hence, we cannot exclude that the absence of caffeine in the tested product may have had an additional positive role in the study.

## 5. Conclusions

Body weight reduction is considered the hallmark target to reduce the detrimental effects of MetS on health, and the attainment of this aim requires changes in lifestyle and nutrition [52], promoting exercise, and caloric restriction. The increase of calories expenditures obtained by exercise can be complemented by the use of thermogenic food ingredients, that is, compounds that promote brown adipose tissue activity and, in general, the degradation of fats in a non-ATP producing way. Thermogenic compounds could also provide help in counteracting the metabolic homeostatic adjustments associated to diet, increasing its overall efficacy [53]. The effect of green tea, the best-known dietary thermogenic agent, is therefore expected to be most evident when associated to a lifestyle intervention that involves both dietary restriction and exercise. We have evaluated this possibility in a controlled registry study in borderline MetS individuals, associating a lecithin-formulated decaffeinated catechin extract (GSP) to a controlled hypocaloric diet and exercise schedule. Compared to a control characterized by the same lifestyle intervention scheme, significant beneficial effects were observed not only on weight but also on other markers of cardiovascular and diabetic risk associated with the conditions, making it tempting to speculate that green tea might promote these improvements not only indirectly via weight loss but also directly, owing to its antioxidant and anti-inflammatory activity. We believe that the study we have outlined is worth expanding in terms of both recruitment and duration, also focusing on the chronic subclinical inflammation commonly associated with MetS and obesity. Inflammation strongly contributes to the persistence of the MetS and to the clinical consequences of the syndrome [54–58], and inflammatory markers, often in association with an increased oxidative stress, are usually found in MetS, contributing to its progression to higher risk levels [59].

## Figures and Tables

**Table 1:** Group characteristics at inclusion.

Group	Number of subjects	Men/women ratio	Age (average; ±s.d.; range)	Percentage of subjects with borderline MetS values (for all 5 items)
Treated group (Management plan + MonCam)	50	25/25	47.6 yr.; ±5.5; 45–55	100%
Control group (Management plan + blank formulation)	50	24/26	45.3 yr.; ±3.55; 45–55	100%

**Table 2:** Anthropometric and blood parameters in treated and control groups at the inclusion and at the end of the observation period (24 weeks).

ITEM (inclusion criteria)	Reference for MetS according to NCEP ATP III		Inclusion	24 weeks	*P *
Weight (kg)		GSP	88.4; 2.4	76.6; 2.5	∗ #
		range	83.2–92.5	72.3–82.8	
		control	88.9; 2.1	83.0; 1.8	∗
		range	84.4–91.3	87.0–77.6	
BMI	Normal 18.5 to 25 Overweight 25 to 30	GSP	31.0; 2.0	26.7; 1.7	∗ #
		control	30.9; 2.6	28.9; 2.5	∗
Central obesity (waist circumference)	>102 cm (men) >88 cm (women)	GSP	M 107.0; 1.3 F 91.0; 1.6	95.6; 1.3 82.7; 2.3	∗ # ∗ #
		control	M 105.6; 2.5 F 90.0; 2.4	101; 2.0 88.5; 2.5	∗ ns
Fasting glucose	>110 mg/dL	GSP	116.1; 5.3	107.9; 3.0	∗ #
		control	115.7; 3.7	113.7; 3.0	ns
Triglycerides	>150 mg/dL	GSP	168.0; 8.7	154.7; 7.0	∗ #
		control	163.8; 8.4	160.0; 7.3	∗
HDL cholesterol	M < 40 mg/dL F < 50 mg/dL	GSP	M 34.4; 2.2 F 46.4; 2.5	41.8; 3.3 54.2; 2.2	∗ #
		control	M 36.0; 3.4 F 44.5; 3.4	36.7; 3.2 45.8; 2.0	ns
Plasma free radicals		GSP	466.4; 38	312; 42	∗ #
		control	458.3; 34	439.3; 33	∗
Blood pressure	Systolic BP > 130 mm Hg Diastolic BP > 85 mm Hg	GSP	Sys 136.8; 4.1	129.6; 2.1	∗ #
			Dia 88.8; 2.9	83.6; 2.2	∗ #
		control	Sys 137.4; 3.5	137.5; 2.4	ns
			Dia 89.4; 2.4	86.2; 2.8	ns

*P* < 0.05; *better than inclusion; ^#^better than controls.

**Table 3:** MetS factors presence in 98 subjects (48 treated and 50 controls) at inclusion and at the end of the study.

MetS factors affecting subjects	Inclusion		24 weeks			
	subjects	%	GSP treated (48)		Controls (50)	
			subjects	%	subjects	%
Metabolic syndrome area						
5 over 5	98	100%	1	2%	18	36%
4 over 5	—	—	2	4%	12	24%
3 over 5	—	—	5	10%	10	20%
Borderline						
2 over 5	—	—	7	15%	10	20%
Out of metabolic syndrome area						
1 over 5	—	—	11	23%	0	0%
none	—	—	22	46%	0	0%
